# Sweden’s first Take-Home Naloxone program: participant characteristics, dose endpoints and predictors for overdose reversals

**DOI:** 10.1186/s13011-023-00533-2

**Published:** 2023-04-22

**Authors:** Elin Holmén, Anna Warnqvist, Martin Kåberg

**Affiliations:** 1 Centre for Psychiatry Research, Department of Clinical Neuroscience, Karolinska Institutet, Stockholm, Sweden; 2 Stockholm Needle and Syringe Program, Stockholm Centre for Dependency Disorders, Stockholm, Sweden; 3 Division of Biostatistics, Institute of Environmental Medicine, Karolinska Institutet, Stockholm, Sweden; 4 Department of Global Public Health, Karolinska Institutet, Stockholm, Sweden

**Keywords:** Take-Home Naloxone, People who inject drugs, Needle and syringe program, Opioid overdose, Sweden

## Abstract

**Background:**

Opioid overdoses are a growing concern, particularly among people who inject drugs. Sweden, with a comparatively high proportion of drug-related mortality, introduced its first Take-Home Naloxone (THN) program in 2018, at the Stockholm needle and syringe program (NSP). In this study we compare THN participant characteristics regarding refills and overdose reversals as well as investigate predictors associated with number of reversals. We also investigate interventions performed in overdose situations and endpoints for naloxone doses.

**Methods:**

This was a prospective open inclusion cohort study conducted between January 24^th^ 2018 and March 31^st^ 2022 at the Stockholm NSP. Participants received THN, free of charge, after a training session and provided data regarding drug use and overdose experiences. During refill visits, participants reported if the naloxone was used for overdose reversal and, if so, responded to a ten-item questionnaire which included stating whether the naloxone recipient was the participant themselves or somebody else. Questionnaire data was combined with NSP database demographic data. Zero-inflated Poisson regression was applied to analyse predictors for number of reported overdose reversals.

**Results:**

Among study participants (*n* = 1,295), 66.5% stated opioids as their primary drug, and 61.4% and 81.0% had previous experience of a personal or witnessed overdose, respectively. Overall, 44.0% of participants reported a total of 1,625 overdose reversals and the victim was known to have survived in 95.6% of cases. Stimulant use (aIRR 1.26; 95% CI 1.01, 1.58), benzodiazepine use (aIRR 1.75; 95% CI 1.1, 2.78) and homelessness (aIRR 1.35; 95% CI 1.06, 1.73) were predictors associated with an increased number of reported overdose reversals. Mortality was higher among those who reported at least one overdose reversal (HR 3.4; 95% CI 2.2, 5.2).

**Conclusions:**

An NSP’s existent framework can be utilised to effectively implement a THN program, provide basic training and reach numerous high-risk individuals. During the four-year study, THN participants reversed a sizeable number of potentially fatal overdoses, of which many were reported by participants whose primary drug was not opioids. Naloxone refill rate was high, indicating that participants were motivated to maintain a supply of naloxone in case of future overdose events.

**Supplementary Information:**

The online version contains supplementary material available at 10.1186/s13011-023-00533-2.

## Background

The number of opioid-related overdoses among people who use drugs (PWUD) has increased significantly in recent years, with more than 100,000 fatal opioid overdoses estimated in 2021 in the United States (U.S.) alone [1]. The use of fentanyl and other synthetic opioids has driven the opioid crisis in the U.S. and Canada [2] while heroin remains the most common illicit opioid used in Europe [3].

Drug-related mortality in Sweden rose in the mid-2000s, with opioid overdoses being the most common cause of death [4]. In 2018, the number of fatalities in Sweden decreased slightly, largely due to the reduced availability and use of fentanyl [4]. Despite this, Sweden had the highest proportion of drug-induced deaths in the European Union in 2018 with an estimated 84 deaths per million among adults [3].

While fatal opioid overdoses are the leading cause of death among PWUD, non-fatal overdoses are even more prevalent, especially among people who inject opioids [5, 6]. Non-fatal overdoses are costly for health care systems and can have serious consequences for victims, including hypoxic brain injury with cognitive impairment and memory loss, cardiac failure, peripheral neuropathy and pneumonia [7]. A non-fatal overdose is also a predictor for subsequent fatal drug overdose [6, 8, 9].

Naloxone is an opioid-specific antagonist that efficiently, but temporarily, reverses the acute effects of an opioid overdose thereby preventing potential injuries or fatal outcomes. Naloxone has no potential for abuse and reports of serious adverse effects are scarce, although opioid dependent individuals may experience unpleasant withdrawal symptoms [10]. A significant proportion of PWUD have both witnessed and experienced opioid overdoses at some point in life [11, 12] and in heroin related overdoses most fatalities occur 20–30 min after use, providing a window for naloxone interventions [13].

Naloxone was initially used as an emergency treatment for opioid overdose by health care personnel, but increased overdose numbers led to the initiation of the first Take-Home Naloxone (THN) programs in the U.S. in 1996 [14]. THN programs distribute naloxone directly to individuals at risk of experiencing or witnessing an opioid overdose, with the aim of a prompt initiation of overdose reversal in order to reduce mortality and morbidity. Short training sessions have proven to be sufficient to educate lay persons, significantly improving their knowledge of overdose management and safe naloxone administration [15–17]. Broad implementation of THN programs [18] as well as opioid agonist treatment (OAT) [19] and safer drug consumption sites [20] have been identified as key strategies that can reduce the number of fatal opioid overdoses.

Previous quantitative studies on THN programs have included cost effectiveness [21], level of training needed [15] and characteristics associated with naloxone refill and overdose reversals [22, 23]. A recent Swedish study compared the uptake and use of THN in OAT and needle and syringe program (NSP) populations and concluded that NSP clients constituted a high-risk group that was more likely to report overdose reversals [24]. Other Nordic research has focused on the role of “super users”, i.e. people who have reported more than three overdose reversals, highlighting young age, heroin use and prior overdose experience as significant characteristics of this population [25].

In Sweden, it took several years to overcome regulations that hampered the introduction of THN programs and when the National Board of Health and Welfare finally approved THN implementation in June 2017, naloxone was not made available for THN programs until the following year. While widespread in some parts of the country [26], THN cover is patchy elsewhere [27].

Policy and legal barriers continue to prevent THN programs from fulfilling their full potential to save lives [14]. Sweden lacks a national government-funded THN program, something which its neighbour Norway has had since 2014 [28]. According to Swedish legislation, THN prescription is personal and restricted to individuals at risk of an overdose, it must also be combined with information and basic training. Consequently, the family or friends of a person at risk cannot be prescribed naloxone [29], however the actual overdose training material can be accessed online by family members and other potential bystanders [30], or via some regional initiatives [26]. Considering these limitations, current THN programs need to be assessed in order to further inform health care decisions and policy makers.

In this study we use data from Sweden’s first official THN program which was introduced at the Stockholm NSP in 2018. The study sample consists of people who inject drugs (PWID), a high-risk group per se, in a setting where a high prevalence of opioid overdoses was anticipated. The aim of the study was to compare THN participant characteristics regarding naloxone refill and reports of overdose reversal and to further investigate:

1. Predictors associated with the number of reports of overdose reversal among THN participants.
2. Overdose situations and interventions performed, when THN was used.
3. Naloxone endpoints i.e. what happened to the individual naloxon doses given out in the THN program.

## Methods

### Study setting

The Stockholm NSP opened in April 2013 and consists of two clinics and one mobile unit. To date, more than 4,300 individuals have been registered in the program. The NSP offers sterile injection equipment such as needles, syringes and other paraphernalia (filters and cookers). In addition, services include THN and overdose prevention training, vaccination, counselling, wound care, treatment for hepatitis C (HCV) and HIV as well as referrals to other service providers for OAT and other substance use disorder treatments. In accordance with Swedish legislation, eligibility criteria for NSP enrolment include current injection drug use, being 18 years of age or older, and being able to prove your identity. Testing for hepatitis A, hepatitis B, HCV and HIV is offered on enrolment and continuously. The unique Swedish personal identity number is used for registration and those without such an ID are provided with a unique reserve number.

Study inclusion criteria were THN program enrolment in the Stockholm NSP and at least one additional NSP visit during the study period.

### Study design and THN intervention

This was a prospective open inclusion cohort study conducted between January 24^th^ 2018 and March 31^st^ 2022. Clients in the Stockholm NSP were informed and recruited to the THN program through personal information from NSP staff, also promoted on an information screen in the waiting room. The study design relied on the existing NSP structure with passive follow-up when participants returned to the NSP for regular visits, there was no active following-up outside of the NSP. During the study period’s first four months, the only available naloxone on the Swedish market was a prefilled vial for intra muscular (i.m.) administration (Prenoxad, 0.4 mg/ml) which contained five doses of naloxone. From May 2018, i.m. naloxone was gradually replaced within a few months by an intranasal solution (Nyxoid, 1.8 mg/dose) which was given out in a kit containing two doses.

All THN participants individually completed a single training session, carried out by a NSP nurse or physician, before naloxone was handed out the first time. The sessions were brief (10–15 min) and conducted on the premises of the NSP clinics. A refresher training session was offered on-demand when returning for a refill of naloxone. The training protocol was based on the European Monitoring Centre for Drugs and Drug Addiction’s (EMCDDA) recommendations [31] and included information on how to identify an overdose, general overdose response, basic cardiopulmonary resuscitation (CPR) with focus on rescue breathing, and how to administer the naloxone (i.m. or nasal). Participants were informed of possible side effects, including the risk of withdrawal symptoms, the half-life of naloxone and the importance of calling for an ambulance in an overdose situation. At the end of the session, participants were given the opportunity to practice their skills on a CPR dummy and received a prefilled vial of naloxone for i.m. use, or two doses of nasal naloxone, free of charge. Participants were advised to return for a refill and debriefing if their current dose was used for an overdose reversal, lost, expired, given away or in any other way went missing. Participants did not receive any renumeration for participation in study.

THN participants were offered a pocket-sized brochure with key messages from the training. One year into the project, the National Board of Health and Welfare published standardised material concerning THN programs and overdose prevention, which was then distributed in the training sessions [30].

### Questionnaires and definitions of measurements

All study data was registered in the national quality register InfCare Needle Syringe Program (InfCare NSP) database, previously described in detail [32]. Six InfCare NSP questionnaires were used for this study: 1) the ‘NSP enrolment questionnaire’: basic socio-demographic data and drug history; 2) the ‘NSP standard visit questionnaire’: at every NSP visit, clients report which drug they last injected; 3) the ‘Three-to-six-month NSP follow up questionnaire’: updated information on employment and housing status; 4) the ‘Twelve-month NSP follow-up questionnaire’: updated information on employment, housing status and primary drug the past 12 months; 5) the ‘THN enrolment questionnaire’: primary drug and previous experiences of drug overdose; and 6) the ‘THN refill questionnaire’: information on what happened to the previous naloxone dose and, if naloxone was used to reverse an overdose, ten follow-up questions on the intervention.

THN questions were partly adapted from the Norwegian THN training curriculum [33].

Overdose reversals were reported by the person whose prescribed naloxone dose had been used in the event. Consequently, the person experiencing the overdose could have been the study participant themselves or somebody else.

The latest available information was presented for variables concerning housing, education level, income type and primary drug. Static baseline variables included: age at inclusion in THN program, gender, country of birth, and experience of personal or witnessed overdose at inclusion in THN program. Housing status was re-coded into three main categories: homeless (which included sleeping in tents, in garages, in night shelters, on friends’ sofas and so on); unstable housing (temporary housing like hostels, rehabilitation homes etc.) and stable housing (longer-term rental or own home).

For intramuscular naloxone (which was only distributed in the first few months of the program), although the vial technically contained five doses, it was treated as a single dose in reporting.

Data on mortality were automatically reported from the Swedish population registry to the Stockholm NSP medical chart and quality register.

### Statistics

Continuous variables were presented both as mean (standard deviation) and median (inter quartile range) values, and categorical variables as counts and percentages. Between-group differences were presented using risk ratios (RR) for categorical variables and mean and median differences for continuous variables. RR were estimated using a log-linear model and mean and median differences using linear and quantile regression respectively.

The association of baseline information and number of naloxone doses used at overdose was estimated in Incidence Rate Ratios (IRR) using a zero-inflated Poisson regression model, offsetting for the time participants spent in the study (time-in-study). The offset was applied due to the high variance of time-in-study which directly affected the probability of reporting overdose reversal. Univariable models as well as a multivariable model with all covariates were estimated. All variables from the unadjusted model were included in the adjusted model (gender, age, country of birth, housing, primary drug, and overdose experience). The logistic zero-inflation part of the model only used the number of naloxone doses received as an independent variable, while the independent variables in the Poisson part of the model were changed depending on the variable of interest.

The association between background information and the endpoint of individual naloxone doses (i.e. was it used, lost, stolen etc.) was estimated in Relative Risk Ratios (RRR) using a multinomial logistic regression model. The clustered robust standard error estimator was used to account for repeated measures since a person could report different endpoints multiple times due to receiving more than one refill.

The difference in mortality between the group of participants that had reported at least one overdose reversal compared to the group that had not, was estimated in Hazard Ratios (HR) using an illness-death model based on the Cox proportional hazards model.

Due to very low levels of missing data, we restricted the analysis to subjects with complete data on the variables involved. Confidence interval (CI) level was set at 95%. All reported *p*-values are two-sided and a *p*-value of < 0.05 was considered as statistically significant.

Analysis was done in Stata, version 15 (StataCorp. 2017. Stata Statistical Software: Release 15. College Station, TX: StataCorp LLC).

## Results

### Participants and characteristics

**Fig. 1:**
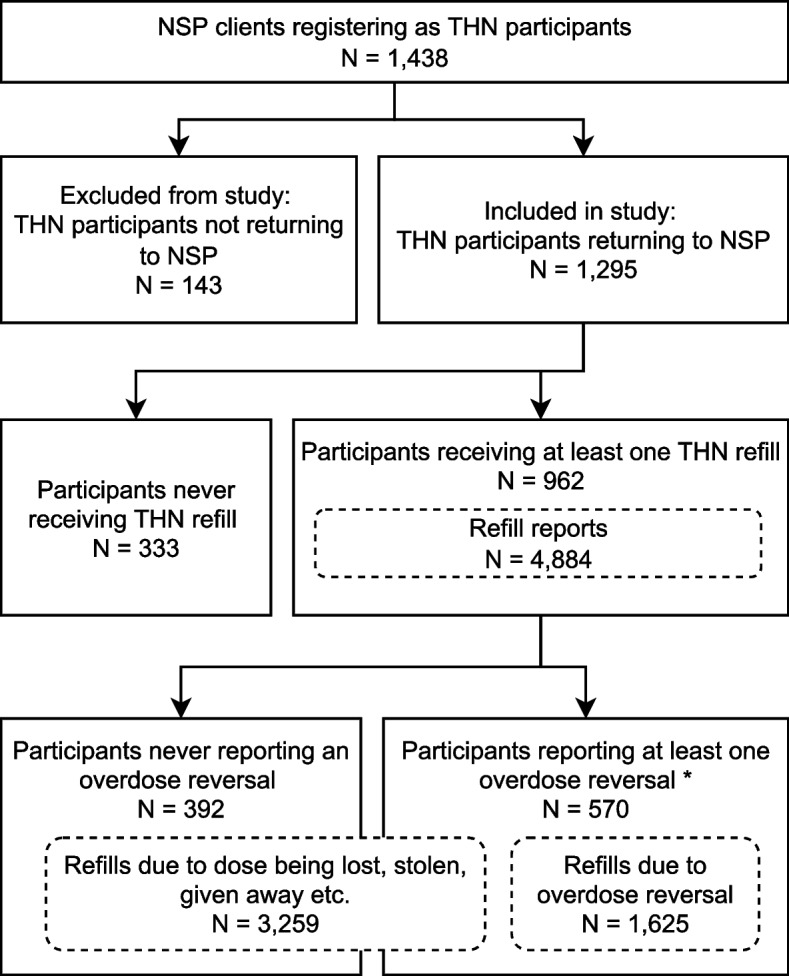
Flowchart of THN participants * Since participants could make multiple refill reports, some of the reports they submitted were for doses not used for overdose reversal

During the study period, 3,151 individuals visited the Stockholm NSP and 1,438 of them enrolled in the THN program. After excluding 143 people who did not make a return visit to the NSP during the study period, the final analysis contained 1,295 individuals. Naloxone was used in 1,625 separate overdose events, reported by 570 individuals, during the four-year study period (Fig. 1). A total of 11,440 doses of naloxone were distributed (2,590 at enrolment and 8,850 at subsequent refills).

The mean age of participants was 38, with the majority being male and born in Sweden, in line with the general population in the Stockholm NSP [32]. The majority stated opioids (mostly heroin) as their primary drug and had prior experience of personal as well as witnessed overdoses (Table 1).

**Table 1:** Characteristics of Take-Home Naloxone participants by refill and reversal status (*N* = 1,295)

Variable	All	No Refill	Refill received	RR (CI)	*P*-value	No Reversal	Reversal reported	RR (CI)	*P*-value
	*N* = 1,295	*N* = 333	*N* = 962			*N* = 392	*N* = 570		
**Gender**	N (%)	N (%)	N (%)			N (%)	N (%)		
Man	951 (73.4)	256 (76.9)	695 (72.2)	1 (ref)	-	266 (67.9)	429 (75.3)	1 (ref)	-
Woman	344 (26.6)	77 (23.1)	267 (27.8)	1.06 (0.99, 1.14)	0.09	126 (32.1)	141 (24.7)	0.86 (0.75, 0.97)	< .02
**Age at inclusion**									
Mean (SD)	38.0 (11.3)	39.8 (11.8)	37.4 (11.0)	-2.4 (-3.8, -1.01)	0.001	37.9 (11.3)	37.0 (10.7)	-0.87 (-2.29, 0.54)	0.23
Median (IQR)	36 (29–46)	39 (30–50)	35.5 (29–45)	-3 (-5.19, -0.81)	< .01	36 (29–46)	35 (29–44)	-1 (-2.96, 0.96)	0.32
**Country of birth**									
Sweden	984 (76.0)	248 (74.5)	736 (76.5)	1 (ref)	-	305 (77.8)	431 (75.6)	1 (ref)	-
Rest of Europe	109 (8.4)	34 (10.2)	75 (7.8)	0.92 (0.81, 1.05)	0,21	35 (8.9)	40 (7.0)	0.91 (0.73, 1.14)	0.41
Rest of the World	150 (11.6)	42 (12.6)	108 (11.2)	0.96 (0.87, 1.07)	0.48	36 (9.2)	72 (12.6)	1.14 (0.98, 1.32)	0.08
Missing	52 (4.0)	9 (2.7)	43 (4.5)	-	-	16 (4.1)	27 (4.7)	-	-
**Housing situation**									
Stable	451 (34.8)	139 (41.7)	312 (32.4)	1 (ref)	-	132 (33.7)	180 (31.6)	1 (ref)	-
Unstable	532 (41.1)	124 (37.2)	408 (42.4)	1.11 (1.03, 1.20)	< .01	169 (43.1)	239 (41.9)	1.02 (0.90, 1.15)	0.81
Homeless	238 (18.4)	52 (15.6)	186 (19.3)	1.13 (1.03, 1.24)	< .01	69 (17.6)	117 (20.5)	1.09 (0.94, 1.26)	0.25
Other	68 (5.3)	17 (5.1)	51 (5.3)	1.08 (0.93, 1.26)	0.29	20 (5.1)	31 (5.4)	1.05 (0.83, 1.34)	0.67
Missing	6 (0.5)	1 (0.3)	5 (0.5)	-		2 (0.5)	3 (0.5)	-	-
**Income type**									
Stable	207 (16.0)	57 (17.1)	150 (15.6)	1 (ref)	-	66 (16.8)	84 (14.7)	1 (ref)	-
Irregular	1,040 (80.3)	262 (78.7)	778 (80.9)	1.03 (0.94, 1.13)	0.49	311 (79.3)	467 (81.9)	1.07 (0.92, 1.25)	0.37
Other	40 (3.1)	12 (3.6)	28 (2.9)	0.97 (0.78, 1.20)	0.76	12 (3.1)	16 (2.8)	1.02 (0.72, 1.45)	0.91
Missing	8 (0.6)	2 (0.6)	6 (0.6)	-	-	3 (0.8)	3 (0.5)	-	-
**Education level**									
< 9 years	138 (10.7)	29 (8.7)	109 (1.3)	1 (ref)	-	42 (10.7)	67 (11.8)	1 (ref)	-
9 years	554 (42.8)	141 (42.3)	413 (42.9)	0.94 (0.85, 1.04)	0.25	162 (41.3)	251 (44.0)	0.99 (0.84, 1.17)	0.9
> 9 years	576 (44.5)	156 (46.8)	420 (43.7)	0.92 (0.84, 1.02)	0.12	182 (46.4)	238 (41.8)	0.92 (0.78, 1.09)	0.35
Missing	27 (2.1)	7 (2.1)	20 (2.1)	-	-	6 (1.5)	14 (2.5)	-	-
**Primary drug**									
Opioids	861 (66.5)	206 (61.9)	655 (68.1)	1 (ref)	-	255 (65.2)	400 (70.3)	1 (ref)	-
Stimulants	372 (28.7)	109 (32.7)	263 (27.3)	0.93 (0.86, 1.0)	0.06	118 (30.2)	145 (25.5)	0.90 (0.80, 1.02)	0.11
Benzodiazepines	34 (2.6)	4 (1.2)	30 (3.1)	1.16 (1.02, 1.32)	0.02	13 (3.3)	17 (3.0)	0.93 (0.67, 1.28)	0.65
Other	23 (1.8)	11 (3.3)	12 (1.2)	0.69 (0.46, 1.02)	0.06	5 (1.3)	7 (1.2)	0.96 (0.59, 1.55)	0.59
Missing	5 (0.4)	3 (0.9)	2 (0.2)			1 (0.3)	0 (0.0)	-	-
**Ever experienced personal overdose**									
No	454 (35.1)	134 (40.2)	320 (33.3)	1 (ref)	-	168 (42.9)	152 (26.7)	1 (ref)	-
Yes	795 (61.4)	183 (55.0)	612 (63.6)	1.09 (1.02, 1.17)	< .02	213 (54.3)	399 (70.0)	1.37 (1.21, 1.56)	< .001
Missing	46 (3.6)	16 (4.8)	30 (3.1)	-	-	11 (2.8)	19 (3.3)	-	-
**Most recent personal overdose**									
< 12 months ago	340 (42.8)	69 (37.7)	271 (44.3)	1 (ref)	-	85 (39.9)	186 (46.6)	1 (ref)	-
> 12 months ago	442 (55.6)	114 (62.3)	328 (53.6)	0.93 (0.86, 1.01)	0.07	123 (57.7)	205 (51.4)	0.91 (0.81, 1.02)	0.11
Missing	13 (1.6)	0 (0.0)	13 (2.1)	-	-	5 (2.3)	8 (2.0)	-	-
**Ever witnessed overdose**									
No	210 (16.2)	61 (18.3)	149 (15.5)	1 (ref)	-	82 (20.9)	67 (11.8)	1 (ref)	-
Yes	1,049 (81.0)	261 (78.4)	788 (81.9)	1.06 (0.96, 1.16)	0.23	301 (76.8)	487 (85.4)	1.37 (1.14, 1.66)	0.001
Missing	36 (2.8)	11 (3.3)	25 (2.6)	-	-	9 (2.3)	16 (2.8)	-	-
**Most recent witnessed overdose**									
< 12 months ago	562 (53.6)	118 (45.2)	444 (56.3)	1 (ref)	-	132 (43.9)	312 (64.1)	1 (ref)	-
> 12 months ago	465 (44.3)	136 (52.1)	329 (41.8)	0.90 (0.83, 0.96)	< .01	162 (53.8)	167 (34.3)	0.72 (0.64, 0.82)	< .001
Missing	22 (2.1)	7 (2.7)	15 (1.9)	-	-	7 (2.3)	8 (1.6)	-	-
**Time in study**									
Mean days (SD)	637.7 (470.0)	319.1 (344.7)	747.9 (456.7)	1.00 (1.00, 1.00)	< .001	639.0 (427.4)	822.8 (461.5)	1.00 (1.00, 1.00)	< .001

*RR* Risk Ratio, *CI* Confidence Interval

By the end of the four-year study period, the majority of participants (74.3%) had returned to the NSP for at least one refill of naloxone and 44.0% had reported that their naloxone dose had been used in at least one overdose situation. Participants receiving a refill were more likely to: be younger, be homeless or in unstable housing, use benzodiazepines as their primary drug, have previous experience of personal overdose, or have a longer time-in-study. Reporting at least one overdose reversal with naloxone during the study period was associated with previous experience of personal or witnessed overdose, being male, and longer time-in-study. The comparison between participants receiving a refill or not, as well as participants reporting reversal or not, is depicted in Table 1.

Overall, 8.6% of the participants reporting reversals died during the study period compared to 4.1% within the group of participants not reporting reversals. The Cox regression analysis showed that the mortality was significantly higher among those who reported a reversal (HR 3.4; CI 2.2, 5.2).

### Predictions of naloxone and number of reversals

We used a zero-inflated Poisson regression to evaluate predictors of number of reported overdose reversals among participants who received at least one refill. In the adjusted model, we noted a greater average number of reported reversals by homeless participants (aIRR 1.35; CI 1.06, 1.73) compared to those with other housing situations. Those with benzodiazepines (aIRR 1.75; CI 1.1, 2.78) or stimulants (aIRR 1.26; CI 1.01, 1.58) as their primary drug reported a greater average number of reversals than those who primarily used other drugs. Additionally, participants who were born outside of Europe (aIRR 1.37; CI 1.06, 1.76) also reported a higher average number of reversals than those born in Europe (including Sweden). The result of the unadjusted and adjusted model is summarized in Table 2.

**Table 2:** Unadjusted and adjusted Zero-inflated Poisson multivariate model predicting naloxone reversal count among participants obtaining refill (*N* = 962)

	**Unadjusted**			**Adjusted**		
**Variables**	**IRR**	**95% CI**	***P*-value**	**aIRR**	**95% CI**	***P*-value**
**Gender**						
Man	1 (ref)	-	-	1 (ref)	-	-
Woman	1.08	0.88, 1.33	0.45	1.08	0.87, 1.33	0.33
**Age at inclusion**						
Mean	1.0	0.99, 1.01	0.79	1.0	0.99, 1.01	0.61
**Country of birth**						
Sweden	1 (ref)	-	-	1 (ref)	-	-
Rest of Europe	0.89	0.69, 1.15	0.37	0.94	0.72, 1.23	0.63
Rest of the World	1.26	0.99, 1.61	0.06	1.37	1.06, 1.76	0.02
**Housing situation**						
Stable	1 (ref)	-		1 (ref)	-	
Unstable	1.1	0.91, 1.33	0.32	1.1	0.89, 1.34	0.38
Homeless	1.34	1.05, 1.72	0.02	1.35	1.06, 1.73	0.02
Other	0.84	0.60, 1.18	0.32	0.89	0.64, 1.24	0.48
**Primary drug**						
Opioids	1 (ref)	-		1(ref)	-	
Stimulants	1.14	0.92, 1.42	0.24	1.26	1.01, 1.58	0.04
Benzodiazepines	1.46	0.89, 2.42	0.14	1.75	1.1, 2.78	0.03
Other	1.12	0.54, 2.35	0.76	1.38	0.72, 2.68	0.33
**Ever experienced personal overdose (baseline)**						
No	1 (ref)	-		1(ref)	-	
Yes	1.14	0.93, 1.39	0.2	1.19	0.97, 1.47	0.10
**Ever witnessed overdose (baseline)**						
No	1 (ref)			1(ref)		
Yes	1.3	1.00, 1.69	< 0.05	1.27	0.95, 1.71	0.10

*IRR* Incidence Rate Ratio, *CI* Confidence Interval

### Overdose situations

In the case of overdose reversals, the majority of refills (63.2%) were requested within four weeks of the incident (Table 3). In most cases (67%), overdose reversals were reported to have been carried out on an acquaintance. Just over half (51.8%) of the overdoses took place in a private space (normally the participant’s or somebody else’s home) while 45.3% occurred in a public space (most frequently “outdoors”). Homeless participants had more than twice the risk of reporting that the reversal took place in a public space than participants who were not homeless (RR 2.42; CI 1.60, 3.67).

**Table 3:** Characteristics of overdose situations where THN was used (*N* = 1,625)

Variable	N (%)
**Who was the recipient of naloxone?**	
Me	264 (16.2)
Stranger	149 ( 9.2)
Partner/Spouse	100 ( 6.2)
Friend/acquaintance	1,089 (67.0)
Missing	23 ( 1.4)
**Number of naloxone doses administered at overdose**	
1	779 (47.9)
2	663 (40.8)
> 2	71 (4.4)
Missing	112 (6.9)
**When did the overdose take place?**	
Less than a week ago	401 (25.4)
1–4 weeks ago	626 (39.6)
1–3 months ago	311 (19.7)
4–6 months ago	132 (8.4)
7–12 months ago	74 (4.7)
Over a year ago	35 (2.2)
**Where did the overdose take place?**	
Private space	802 (51.8)
Public space	701 (45.3)
Shelter	36 (2.3)
Other	8 (0.5)
**Was CPR given?**	
No	1,057 (65.0)
Yes	568 (35.0)
**Was an ambulance called?**	
No	868 (53.4)
Yes	757 (46.6)
**If an ambulance was called, was the naloxone recipient taken to hospital?**	
No	218 (28.8)
Yes	423 (55.9)
Other	16 (2.1)
Don’t know	100 (13.2)
Missing	3 (0.2)
**What drugs were believed to be used prior to the overdose? (multiple answers possible)**	
Opioids	1,537 (94.6)
Stimulants	97 (6.0)
Benzodiazepines	565 (34.8)
Other	128 (7.9)
**Did the naloxone recipient survive?**	
Yes	1,539 (95.6)
No	8 (0.5)
Unknown	59 (3.7)
**How comfortable do you feel administering naloxone?**	
Very comfortable	1,167 (73.4)
Quite comfortable	314 (19.7)
Only slightly comfortable	11 ( 0.7)
Not at all comfortable	5 ( 0.3)
Don’t know	18 ( 1.1)
Missing	76 ( 4.8)

Apart from administering naloxone, overdoses were responded to with actions promoted in the overdose training such as rescue breathing and/or heart compressions (35%) or calling an ambulance (46.6%) (Table 3). The vast majority of participants reported that opioids (on their own or in combination with another drug) were believed to have been used prior to the overdose (Table 3). Apart from opioids, benzodiazepines were the most common additional drugs involved, reported in 34.8% of cases.

Participants reported that the person who experienced an overdose was known to have survived in 95.6% of incidents. There were eight cases (0.5%) where the person who experienced an overdose could not be resuscitated and subsequently died. The outcome of the remaining cases was unknown. The vast majority (93.1%) of the participants responded that they felt very comfortable, or quite comfortable, using naloxone in overdose situations.

### Naloxone dose endpoints

THN participants made a total of 4,884 refill reports, stating that the previous dose was used in an overdose situation in 33.3% of these reports. Other reasons for refill were: dose lost (32.9%), dose given away (15.5%), dose stolen (4.9%) or “other reason” (13.3%, most commonly the previous dose had expired or been confiscated by police or security guards). We used multinomial logistic regression to explore participant predictors for different naloxone dose endpoints (Supplementary Tables S1a-c). The risk of giving away the naloxone dose was higher among participants with stimulants as their primary drug (RRR 1.46; CI 1.07, 1.98) compared to those who primarily used other substances, and lower amongst those with prior experience of personal overdose (RRR 0.60; CI 0.50, 0.88) or witnessed overdose (RRR 0.66; CI 0.41, 0,85) compared to participants with no such experiences. Having the dose stolen was more likely amongst participants who were homeless (RRR 3.73; CI 2.06, 6.75) or in unstable housing (RRR 1.9; CI 1.03, 3.42) compared to people with other housing situations; those whose primary drug was stimulants (RRR 1.77; CI 1.14, 2.74) compared to those who mainly used other substances; women (RRR 1.70; CI 1.17, 2.47) compared to men; and participants with prior experience of witnessed overdose (RRR 2.18; CI 1.12, 4.23) compared to those without such experience. Participants with experience of personal overdose on the other hand, were less likely to report this outcome (RRR 0.51; CI 0.34, 0.76). Lastly, the risk of losing the naloxone dose was higher among those with unstable housing (RRR 1.32; CI 1.04, 1.68) and homelessness (RRR 1.91; CI 1.47, 2.48) compared to participants with other housing situations and lower amongst those with personal (RRR 0.64; CI 0.50, 0.81) and witnessed overdose experience (RRR 0.65; CI 0.45, 0.94) compared to those without such experiences.

## Discussion

In this study we investigated the first Take-Home Naloxone program introduced in Sweden, presenting demographic and behavioural characteristics associated with naloxone refill and reporting reversals as well as overdose situations and endpoints for distributed naloxone kits. We noted that over two thirds of the participants returned to the NSP for a refill during the study period, a higher proportion than reported in previous research [24, 34–36].

Overall, 44% of participants reported their naloxone being used to reverse an overdose at least once during the four-year long study period, totalling 1,625 overdose reversals. This is a large proportion compared to some other studies with similar passive follow‐up designs, which stated lower figures of between 7 and 20% of PWUD accessing THN programs reporting at least one of their doses being used for reversal [22, 24, 34, 36–39]. While this comparison may be tempered by differences in settings (such as prevalence of fentanyl) and the duration of the studies, the high number of reported reversals in our study suggests a high-risk study population in the NSP, and that experiencing and witnessing non-fatal overdoses was strikingly prevalent for THN program participants in Stockholm.

The majority of participants requested a new dose within four weeks of having used naloxone in an overdose event, implying that participants were motivated to maintain their naloxone supply and a low threshold for naloxone refill at the NSP. Although naloxone carriage rate was not specifically investigated in this study, previous research emphasises the importance of generous access to new doses in order to increase the chance of having naloxone accessible in critic situations [40–42].

The comparison between participants who reported overdose reversals and those who did not generally confirmed findings from previous studies on THN programs: that being a man [34] and prior experience of personal or witnessed overdose [22–24, 34, 43, 44] are factors associated with reporting overdose reversals. An unexpected finding in our study was that participants who stated stimulants or benzodiazepines as their primary drug reported a significantly greater average number of overdose reversals compared to people using opioids. This supports the argument for widespread distribution of naloxone to PWID who may not see themselves primarily as opioid users, also highlighted by Rowe et al. [22].

The data on which drugs were taken immediately before the overdose were self-reported by participants, and no information was collected regarding contamination of drugs. Swedish healthcare systems should consider its own readiness for sudden changes to the drug market, informed by experiences in other countries such as the impact of synthetic opioids on the U.S. and Canada [2] and how contaminated benzodiazepines have fuelled an epidemic of drug related deaths in Scotland [45]. Sudden changes to the supply of illegal drugs in Sweden will require the ability to promptly scale-up naloxone distribution [46].

Although the level of overdose reversals in our study was high, the majority of naloxone refills were not related to naloxone having been used in overdose situations. People with an unstable housing situation may face challenges in storing personal belongings, including naloxone, and as a result they had an increased risk of their dose being lost or stolen. Additionally, women were more likely than men to have their naloxone dose stolen, supporting previous research in the Stockholm NSP highlighting women’s vulnerability among PWID [47].

A current major challenge for increased access to naloxone in Sweden is that the national legislation is incompatible with prescribing naloxone to anyone other than the person at risk of overdosing [29]. In our study, 15.4% of the refills were due to the naloxone being given away to friends and family members, which pinpoints an unmet need for naloxone distribution. Further, our data show that often the person who overdosed was not the person who had initially been prescribed naloxone, demonstrating that current Swedish legislation concerning THN programs does not reflect the needs of this population.

Also, during the implementation of the THN program, there were frequent requests to the NSP for naloxone from the public, police, security guards, social workers and shelter staff, but current Swedish legislation prevents THN programs from meeting this demand. The inability to supply potential overdose responders with THN is thus a barrier for ensuring the availability of naloxone when and where it is needed. Changing current legislations and making naloxone available over-the-counter are strategies that could increase access for potential bystanders [48].

In the training sessions at the Stockholm NSP, participants were always advised to call for an ambulance and to remain with the person who had overdosed. In our data, an ambulance was called to the scene in 46.6% of the overdoses, which is similar to comparable research [18, 22, 24, 38]. Numerous international studies support the idea that PWUD refrain from calling an ambulance due to fear of police involvement [49–52], losing custody of children or risk of eviction [53–55]. This could well explain why participants did not call an ambulance, since PWUD in Sweden are at risk of being arrested when seeking medical attention in overdose situations as both use and possession of illicit drugs are criminalised. This requires further investigation.

We also observed that 45.3% of reported overdoses took place in a public space. Public drug use may lead to riskier injection practices when individuals are rushed to inject in unsafe environments [56]. International studies conclude that supervised drug consumption sites greatly benefit PWUD, including preventing premature mortality [20]. The high level of overdoses in our study indicates that establishing such sites would provide a safer and clean environment for drug use, especially benefiting PWID living under unstable housing conditions.

We found a higher mortality among those who reported overdose reversals compared to those who did not, which may be related to the high-risk characteristics of this population, i.e. being a man or having a high level of previous overdose experience [8, 57]. However, current lack of information on causes of death among Stockholm NSP clients complicates interpretation of mortality data and requires further study.

Evaluating possible effects of COVID-19 on the study, which impacted half-way through, is not within the scope of this analysis. However, previous research conducted in the Stockholm NSP during the first year of COVID-19, showed that naloxone distribution remained at pre-pandemic levels [58, 59].

A major strength of this study is the large study sample along with linking data to participants’ unique personal identity number, providing opportunity to follow individual participants prospectively.

There are also several limitations. Questionnaires were based on self-reported data which carries the risk for recall- or social desirability bias. The passive follow-up study design relying on spontaneous requests for a refill by THN participants, might lead to misclassification of participants, which may result in underestimation of the number of participants who report naloxone being used, also noted by Siegler et al. [60]. However, our results are strengthened by the many reports of overdose reversals and a large number of refills within a reasonable follow-up time (65.0% within four weeks and 84.7% within three months).

Restricting drug use classification to a primary drug prevented recording information on poly drug use. This limits the conclusions regarding the association between drug use and reports of overdose reversals. This limitation was partly mediated by the study’s method of relating the refill report to the participant’s most recent response on primary drug, capturing changes to an individual’s primary drug use over time.

In accordance with Swedish legislation, clients who enrol in the NSP must verify their identity, as anonymity is not allowed, which is a potential barrier to participation [47]. As a consequence, the reach of the THN program within the larger community of people who are at risk of opioid-overdose is not known, further complicated by the lack of reliable data on overall numbers of PWID in Stockholm. Lastly, since our study population only represent NSP clients, our results may not fully reflect PWID outside an NSP setting.

## Conclusions

This study adds to the scarce data on THN programs in Sweden and concludes that the existing framework of an NSP can be utilised to effectively implement a THN program, provide basic training and reach a large number of high-risk individuals. During the four-year study period, THN participants confidently reversed a sizeable number of potentially fatal overdoses, many of which were a result of access to naloxone among participants not primarily using opioids. The rate of naloxone refills was high, indicating that participants were motivated to maintain a supply of naloxone in case of future overdose events.

Current THN programs in Sweden should be expanded to ensure that naloxone is available whenever needed by modifying the restrictions on who can be prescribed or access naloxone. THN programs also need to be supported by a broader and consistent approach to harm reduction where policymakers could consider new interventions such as safer drug consumption sites in order to reduce the risk of overdose fatality.

## Supplementary Information

**Additional file 1: Table S1a.** Adjusted multinomial logistic regression showing naloxone dose endpoints: Dose lost compared to used for overdose reversal. Table S1b. Adjusted multinomial logistic regression showing naloxone dose endpoints: Dose given away compared to used for overdose reversal. Table S1c. Adjusted multinomial logistic regression showing naloxone dose endpoints: Dose stolen compared to used for overdose reversal.

## Data Availability

The datasets used by the study are available from the corresponding author on reasonable request.

## Abbreviations

CI: Confidence interval
CPR: Cardio pulmonary resuscitation
EMCDDA: European Monitoring Centre for Drugs and Drug Addiction
HCV: Hepatitis C virus
HIV: Human immunodeficiency virus
HR: Hazard ratio
I.m: Intramuscular
Inf Care NSP: Inf Care needle syringe program
IRR: Incidence risk ratio
NSP: Needle and syringe program
OAT: Opioid substitution therapy
PWID: People who inject drugs
PWUD: People who use drugs
RR: Risk Ratio
RRR: Relative risk ratio
THN: Take-Home Naloxone
U.S: United States
WHO: World Health Organisation
