# Supplementary material for: Sweden’s first Take-Home Naloxone program: participant characteristics, dose endpoints and predictors for overdose reversals
Source: Subst Abuse Treat Prev Policy. 2023 Apr 22;18:24. doi: 10.1186/s13011-023-00533-2 (PMC10121425; doi:10.1186/s13011-023-00533-2)
Supplement: Supplementary file 1 — Additional file 1: Table S1a. Adjusted multinomial logistic regression showing naloxone dose endpoints: Dose lost compared to used for overdose reversal. Table S1b. Adjusted multinomial logistic regression showing naloxone dose endpoints: Dose given away compared to used for overdose reversal. Table S1c. Adjusted multinomial logistic regression showing naloxone dose endpoints: Dose stolen compared to used for overdose reversal. [file 13011_2023_533_MOESM1_ESM.docx]

**Supplementary tables:**

Table S1a – Adjusted multinomial logistic regression showing naloxone dose endpoints: Dose lost compared to used for overdose reversal (N=1,606)

| **Naloxone endpoint - dose lost** | **RRR** | **95% CI** | **P-value** |
| --- | --- | --- | --- |
|  |  |  |  |
| **Housing situation** |  |  |  |
| Stable | 1 (ref) | **-** | **-** |
| Unstable | 1.32 | 1.04, 1.68 | 0.02 |
| Homeless | 1.91 | 1.47, 2.48 | <.001 |
| Other | 1.17 | 0.74, 1.84 | 0.50 |
| **Witnessed overdose at baseline** |  |  |  |
| No | 1 (ref) | - | - |
| Yes | 0.65 | 0.45, 0.94 | 0.02 |
| **Personal overdose at baseline** |  |  |  |
| **No** | 1 (ref) | - | - |
| **Yes** | 0.64 | 0.50, 0.81 | <.001 |

*RRR=Relative Risk Ratio, CI=Confidence Interval*

*The variables gender, age, housing situation, primary drug and overdose experience were tested, but only variables with significant results are included in the table.*

Table S1b – Adjusted multinomial logistic regression showing naloxone dose endpoints: Dose given away compared to used for overdose reversal (N= 758)

| **Naloxone endpoint -dose given away** | **RRR** | **95% CI** | **P-value** |
| --- | --- | --- | --- |
|  |  |  |  |
| **Primary drug** |  |  |  |
| Opioids | 1 (ref) | - | - |
| Stimulants | 1.46 | 1.07, 1.98 | 0.02 |
| Benzodiazepines | 0.94 | 0.46, 1.81 | 0.86 |
| Other | 0.85 | 0.40, 1.86 | 0.68 |
| **Ever witnessed overdose (baseline)** |  |  |  |
| No | 1 (ref) | - | - |
| Yes | 0.60 | 0.41, 0,85 | <.01 |
| **Ever experienced personal overdose (baseline)** |  |  |  |
| No | 1 (ref) | - | - |
| Yes | 0.66 | 0.50, 0.88 | <.01 |

*RRR=Relative Risk Ratio, CI=Confidence Interval*

*The variables gender, age, housing situation, primary drug and overdose experience were tested, but only variables with significant results are included in the table.*

Table S1c – Adjusted multinomial logistic regression showing naloxone dose endpoints: Dose stolen compared to used for overdose reversal (N=241)

| **Naloxone endpoint - dose stolen** | **RRR** | **95% CI** | **P-value** |
| --- | --- | --- | --- |
|  |  |  |  |
| **Gender** |  |  |  |
| Man | 1(ref) | - | - |
| Woman | 1.70 | 1.17, 2.47 | <.01 |
| **Housing situation** |  |  |  |
| Stable | 1(ref) | - | - |
| Unstable | 1.9 | 1.03, 3.42 | 0.04 |
| Homeless | 3.73 | 2.06, 6.75 | <.001 |
| Other | 1.58 | 0.72, 3.50 | 0.26 |
| **Primary drug** |  |  |  |
| Opioids | 1(ref) | - | - |
| Stimulants | 1.77 | 1.14, 2.74 | 0.01 |
| Benzodiazepines | 0.91 | 0.37, 2.24 | 0.83 |
| Other | 1.81 | 0.45, 7.39 | 0.41 |
| **Ever witnessed overdose (baseline)** |  |  |  |
| No | 1(ref) | - | - |
| Yes | 2.18 | 1.12, 4.23 | 0.02 |
| **Ever experienced personal overdose (baseline)** |  |  |  |
| No | 1(ref) |  |  |
| Yes | 0.51 | 0.34, 0.76 | 0.001 |

*RRR=Relative Risk Ratio, CI=Confidence Interval*

*The variables gender, age, housing situation, primary drug and overdose experience were tested, but only variables with significant results are included in the table.*
